# Effectiveness of a Salivary Testing System to Screen for Periodontal Disease: A Cross-Sectional Study from the NOSE Study

**DOI:** 10.3390/jcm14144965

**Published:** 2025-07-14

**Authors:** Takayuki Kosaka, Shuri Fushida, Masahiro Wada, Tomoya Gonda, Kodai Hatta, Masae Kuboniwa, Arisa Wada, Sumiyo Hashimoto, Hiromi Hatanaka, Makiko Higashi, Takeshi Kikuchi, Keiji Terauchi, Michiko Kido, Yuya Akagi, Kei Kamide, Mai Kabayama, Kazunori Ikebe

**Affiliations:** 1 Department of Removable Prosthodontics and Gerodontology, Graduate School of Dentistry, The University of Osaka, Osaka 565-0871, Japan; fushida.shuri.dent@osaka-u.ac.jp (S.F.); wada.masahiro.dent@osaka-u.ac.jp (M.W.); gonda.tomoya.dent@osaka-u.ac.jp (T.G.); hatta.koudai.dent@osaka-u.ac.jp (K.H.); ikebe.kazunori.dent@osaka-u.ac.jp (K.I.); 2 Department of Preventive Dentistry, Graduate School of Dentistry, The University of Osaka, Osaka 565-0871, Japan; kuboniwa.masae.dent@osaka-u.ac.jp; 3 Nose Town, Osaka 563-0351, Japan; wada.arisa.68@gmail.com (A.W.); sumi.h-0212@docomo.ne.jp (S.H.); houkatu@town.nose.osaka.jp (H.H.); makiko.91129@icloud.com (M.H.); kikutr35@gmail.com (T.K.); kenkou@town.nose.osaka.jp (K.T.); 4 Division of Health Sciences, Graduate School of Medicine, The University of Osaka, Osaka 565-0871, Japan; mkido@sahs.med.osaka-u.ac.jp (M.K.); akagi.yuya.sahs.med@osaka-u.ac.jp (Y.A.); kamide@sahs.med.osaka-u.ac.jp (K.K.); kabayama@sahs.med.osaka-u.ac.jp (M.K.)

**Keywords:** aged, diagnosis, periodontal diseases, saliva

## Abstract

**Background:** This study aimed to evaluate the effectiveness of a saliva-based screening method for periodontal disease among community-dwelling older adults in Japan. **Methods**: A total of 372 study participants (mean age: 73.1 years) with 20 or more remaining teeth were included in the study. Of the six parameters assessed by the Salivary Multi Test (SMT), this study focused on the three parameters related to periodontal disease: occult blood, leukocytes, and proteins. Periodontal tissue examinations were performed based on the Community Periodontal Index (CPI) using partial mouth recording. To evaluate screening accuracy, the sensitivity, specificity, positive predictive value (PPV), and negative predictive value (NPV) were calculated for each of the three markers: occult blood, leukocytes, and proteins. Receiver operating characteristic (ROC) analysis was performed for each SMT item, and area under the curve (AUC) was calculated. Logistic regression analysis was used to calculate the odds ratios for combinations of SMT markers, with the presence of periodontal pockets and gingival inflammation as the respective outcome variables. **Results**: Among the individual markers, occult blood showed the highest diagnostic performance for detecting both periodontal pockets and gingival inflammation. The combination of elevated occult blood and leukocyte levels yielded the highest odds ratios for both periodontal pockets and gingival inflammation. **Conclusions**: While several SMT markers showed associations with periodontal conditions, their utility for screening in older Japanese adults remains to be further validated. Combining markers may help improve diagnostic performance, but additional studies are warranted.

## 1. Introduction

Periodontal disease is a chronic inflammatory disease that causes swelling and pain in the gingiva and destruction of alveolar bone, and it is a major cause of tooth loss in Japanese adults [1]. Periodontal disease progresses with age [2] and is associated with general diseases such as diabetes mellitus [3,4], atherosclerosis [5,6], and cardiovascular disease [7,8], and treatment and prevention of periodontal disease are important for promoting general health. Periodontal disease progresses with slow symptoms, and when symptoms appear, the disease is often well advanced. Therefore, detecting periodontal disease at an early stage and working to control the progression of the disease are necessary [9].

Periodontal pocket depth, bleeding on probing (BOP), and clinical attachment loss (CAL) are commonly used as evaluation methods for periodontal disease [10]. However, these methods are time-consuming, require specialized training, and may vary in reliability across examiners [11], which limits their applicability in large-scale community-based screenings, especially among older adults.

Salivary diagnostics have emerged as promising tools for noninvasive, rapid screening of oral health conditions. Several methods have been developed, such as enzyme activity assays, microbial detection kits, and immunoassays targeting inflammatory biomarkers [12]. Among these, the Salivary Multi Test (SMT) (LION, Tokyo, Japan) offers a unique advantage in simultaneously assessing multiple salivary indicators using a single, user-friendly device [13,14]. Unlike other salivary tests that may require laboratory analysis or extensive equipment, the SMT provides immediate results chairside, making it particularly suitable for elderly individuals who may have difficulty undergoing lengthy oral examinations.

Despite its growing use in preventive dentistry, the utility of the SMT as a screening tool for periodontal disease in community-dwelling older adults remains underexplored. Given the need for practical, scalable screening tools in aging societies, this study aimed to evaluate the usefulness of the SMT by investigating the associations between salivary biomarker levels and clinical periodontal findings in individuals aged 65 years or older in a cross-sectional observational study. This work addresses a gap in current screening strategies by assessing whether the SMT can serve as a feasible alternative to traditional periodontal examination in epidemiologic and primary care settings.

## 2. Materials and Methods

### 2.1. Study Participants

The study participants were 541 community-dwelling older adults (221 men and 320 women) aged 65 years in the NOSE study [15], a prospective, cohort study of preventing the development of cardiovascular disease, frailty, and cognitive decline, from August 2022 to September 2023. Of them, 372 participants (149 men and 223 women, mean age: 73.1 ± 5.0 years) with 20 or more remaining teeth and no missing data in the survey items were included in the analysis. The study was conducted with approval from the ethics review board of Osaka University Graduate School of Dentistry and Osaka University Dental Hospital (Approval No. R4-E7). Only those participants who provided informed consent after receiving a full written and oral explanation of the aims and methods of the study were surveyed. This study conformed to STROBE (Strengthening the Reporting of Observational Studies in Epidemiology) guidelines [16].

### 2.2. SMT Measurement

Prior to oral examinations and periodontal tissue examinations, measurements by the SMT (SY-4911, LION Dental Products, Tokyo, Japan) were performed [13]. SMTs were performed according to the manufacturer’s protocol. The participants were instructed to rinse their mouth with 3 mL of distilled water for 10 s. One examiner dropped a 10 µL sample on each of seven pads of a test strip to analyze the cariogenic bacteria, acidity, buffering capacity, occult blood, leukocytes, proteins, and ammonia. In this study, occult blood, leukocytes, and proteins were used as indicators reflecting periodontal disease. The occult blood level was detected by determining hemoglobin pseudo-peroxidase activity as an index. The leukocyte level was detected by the measurement of esterase activity in isolated salivary leukocyte. The “protein error of indicators” reaction was used to determine protein levels. The color changes on each pad of the test strip were evaluated as reflectivity. The occult blood, leukocytes, and proteins were measured one minute later. The values of the test results were expressed as a relative value (percentage; 0–100% within the detection range) [17].

### 2.3. Periodontal Tissue Examination

Periodontal tissue examinations were performed based on the modified Community Periodontal Index (CPI) using partial mouth recording [18,19]. The teeth examined were the maxillary and mandibular left and right first and second molars, the maxillary right central incisor, and the mandibular left central incisor, for a total of 10 teeth. Periodontal status was examined using a CPI probe (Periodontal Probe; YDM, Tokyo, Japan) at six points in the periodontal pocket of each tooth, and evaluation was performed by recording the highest code according to the following criteria: Code 0, no findings of a periodontal pocket; Code 1, depth of periodontal pocket ≥ 4 mm but <6 mm; and Code 2, depth of periodontal pocket ≥ 6 mm. Participants were classified according to the presence or absence of a periodontal pocket (CPI code ≤ 1/≥ 2) [20]. In addition, the presence or absence of BOP was evaluated. For the number of teeth examined, the number of teeth with BOP was calculated and defined as the BOP rate. Participants with more than the 3rd quartile for the BOP rate, which was 39%, were defined as having gingival inflammation.

### 2.4. Statistical Analysis

The Mann–Whitney U test was used to compare occult blood, leukocyte, and protein levels on the SMT between the groups with different periodontal status. The median values of occult blood, leukocyte, and protein levels on the SMT were used as thresholds to classify the patients into the Normal and Higher groups for each index. The distributions of the Normal group and the Higher group of occult blood, leukocyte, and protein levels between the groups with different periodontal status were compared using the chi-squared test. To evaluate the screening accuracy for periodontal disease, the sensitivity, specificity, positive predictive value (PPV), and negative predictive value (NPV) of occult blood, leucocyte, and protein were determined. In addition, receiver operating characteristic (ROC) analysis was performed for each SMT item, and area under the curve (AUC) was calculated. To examine which two combinations of occult blood, leukocyte, and protein levels best detected the presence of periodontal pockets and gingival inflammation, the distribution of each combination was compared between the groups with different periodontal status using the chi-squared test. In addition, the odds ratio for each combination of SMT items was calculated by logistic regression analysis with the presence of periodontal pockets and gingival inflammation, respectively, as the objective variable adjusting for age and sex. Statistical analysis was conducted using IBM SPSS Statistics version 24 (SPSS Japan Inc., IBM Company, Tokyo, Japan), with the level of significance set at 5%.

## 3. Results

### 3.1. Demographics of Study Participants

Occult blood and protein values were significantly higher in participants with periodontal pockets, and occult blood was also significantly higher in those with gingival inflammation (Table 1). In all SMT items, the Higher group showed a significantly greater proportion of individuals with periodontal pockets, and for occult blood and protein, this was also true for gingival inflammation.

### 3.2. Validity of SMT Measurements

Table 2 summarizes the sensitivity, specificity, PPV, NPV, and AUC for each SMT item. Occult blood showed the highest sensitivity, PPV, NPV, and AUC for diagnosing periodontal pockets; and protein had the highest sensitivity for diagnosing gingival inflammation. Specificity was similar across all markers. However, all AUC values indicated low diagnostic accuracy.

### 3.3. Combinations of SMT Markers

Table 3 shows the associations between combinations of SMT markers and periodontal status. For all marker pairs, the prevalence of periodontal pockets and gingival inflammation was highest when both markers were in the Higher group. Among these, the combination of occult blood and protein showed the strongest association with both conditions.

Logistic regression analysis (Table 4 and Table 5) showed that higher values in any combination of SMT markers were significantly associated with both periodontal pockets and gingival inflammation. The highest odds ratios for both conditions were observed when occult blood and leukocyte levels were elevated.

## 4. Discussion

This study examined the usefulness of the SMT as a screening method for periodontal disease in elderly persons. All SMT items were useful as indicators for the presence of periodontal pockets and gingival inflammation. The combination of high values of both occult blood and protein levels was associated with a greater possibility of the presence of periodontal pockets and gingival inflammation. The results of the present study showed that SMT markers may be useful as screening methods for periodontal disease in elderly persons aged 65 years and older.

Occult blood on the SMT indicates the presence of a small amount of blood in the saliva. A high level of occult blood reflects a high incidence of bleeding in the oral cavity, especially from the gingiva. Leukocytes indicate immune cells in the saliva. A high leukocyte level reflects a high degree of infection or inflammation in the oral cavity. Protein indicates various physiological and pathological conditions in the saliva. A high level of protein reflects high levels of inflammation and stress in the salivary glands. All of these markers are considered to be elevated in the presence of inflammation in the oral cavity, especially with the presence of periodontal disease [21,22]. However, some SMT markers in this study did not show significant results for periodontal disease, because SMT markers measure the amount of reactive substances in the saliva, and thus they do not reflect only the inflammatory response due to periodontal disease, but also various types of inflammation and infection in the oral cavity. These pathogens, which were not evaluated in detail in the present study, may have affected the marker levels.

For periodontal tissue evaluation, periodontal pockets, BOP, and CAL are commonly used [10]. However, in screening large numbers of individuals, an evaluation method that is quick, simple, noninvasive, and nonbiased is required. Saliva has been focused on as a sample that satisfies these conditions and reflects periodontal disease [23,24]. Various salivary markers have been focused on and investigated as indicators of periodontal disease. Kosaka et al. showed, in an epidemiological study of 608 participants, that salivary interleukin-β (IL-1β), IL-6, tumor necrosis factor-α (TNF-α), and prostaglandin E2 (PGE2) levels were correlated with the severity of periodontal disease [25]. Kim et al. reported that algorithms using salivary matrix metalloproteinase-9 (MMP-9) and S100A8 levels showed high diagnostic power for periodontitis [26]. In addition, Relvas et al. found that salivary IL-1β, IL-6, and IL-10 levels were significantly associated with several clinical measurements reflecting periodontal disease [27]. Thus, many studies have reported the usefulness of salivary markers for the diagnosis of periodontal disease. In this respect, the SMT evaluation satisfies the above conditions, and although it is only a screening test, it is useful as a tool for simple and comprehensive evaluation of periodontal disease.

In the present study, periodontal disease was analyzed according to the presence or absence of periodontal pockets ≥6 mm. Early detection and early treatment for periodontal disease are important. The setting of reference values for periodontal pockets should be considered separately according to the purpose of screening and the target population. Periodontal pockets ≥6 mm often indicate advanced destruction of periodontal tissues and require specialized treatment. The criterion of periodontal pockets ≥6 mm is a screening criterion that focuses on severe cases of periodontal disease and identifies patients who should undergo specialized periodontal treatment. In the present study, significant associations were found between SMT markers and the presence of periodontal pockets ≥6 mm. The SMT markers may be useful indicators of severe periodontitis, although more detailed investigations are needed to determine a clear cutoff value for use as a screening method.

In the present study, periodontal disease was evaluated both in terms of the presence or absence of periodontal pockets and BOP. Periodontal pockets are the result of chronic inflammation caused by previously accumulated periodontitis. Therefore, their presence or absence does not necessarily reflect the current inflammatory condition [28]. In contrast, BOP reflects the current gingival inflammatory condition. If the markers of the SMT reflect the inflammatory condition of the oral cavity, we hypothesized that BOP has a stronger association with the SMT markers. However, the mean values for some SMT markers were slightly greater in those with periodontitis than in those with BOP. In the present study, a large percentage (83%) of those with pockets ≥6 mm had BOP, suggesting that a high percentage of those with pockets also had gingival inflammation.

The NPVs of SMT items were relatively high (79% to 90%), suggesting that low SMT values may help to rule out the presence of periodontal pockets or gingival inflammation. This could be a potentially useful feature in the context of simple screening. In contrast, the PPVs were low (22% to 32%), indicating that high SMT values often include false positives and require follow-up clinical evaluation. Among the SMT items, occult blood demonstrated relatively better sensitivity, PPV, and NPV, but this should be interpreted with caution given the modest diagnostic performance. In general, an AUC value between 0.5 and 0.7 is considered low diagnostic accuracy [29]. Therefore, the SMT alone should not be used as a definitive diagnostic tool for periodontal disease.

However, for initial screening purposes, even modest discriminatory ability—if combined with high NPV—may contribute to practical population health strategies, especially in resource-limited or nonclinical settings. Although the SMT is not sufficient alone for definitive diagnosis of periodontal disease, its noninvasiveness and simplicity make it a useful primary screening tool in evaluating the absence of periodontal tissue abnormalities.

Several other salivary diagnostic markers—such as interleukin-1β (IL-1β), matrix metalloproteinase-8 (MMP-8), and bacterial DNA—have also been studied in relation to periodontal disease [30]. However, direct comparisons with the SMT are currently lacking. Future studies should aim to evaluate how the SMT performs relative to these established salivary biomarkers in terms of diagnostic accuracy, feasibility, and cost-effectiveness. These findings suggest that the SMT can serve as an effective initial triage tool in community-dwelling older adults. In particular, the high NPV indicates that individuals with low SMT scores are unlikely to have advanced periodontal disease, thereby reducing the need for unnecessary dental referrals or further examinations. This is especially useful in settings where access to dental professionals is limited, or in populations with reduced mobility or cognitive decline who may not tolerate conventional periodontal assessment. Thus, while the SMT may not replace clinical examinations, it holds practical value in large-scale screening initiatives to prioritize care and optimize resource allocation. However, the relatively high cost of SMT kits may limit their widespread application, particularly in large-scale public health initiatives or settings with limited healthcare resources. Cost-effectiveness studies are needed to determine the feasibility of routine use in population-based screening.

Expecting that the combination of SMT markers would enhance the detection rate of periodontal disease, the associations of combinations of two of occult blood, leukocyte, and protein levels with periodontal disease were analyzed. For any combination, the detection rate of periodontal disease was higher when both of the two markers were high. Furthermore, the detection rate of periodontal disease was higher when the two markers were combined compared with when the association with periodontal disease was examined using a single marker alone. When screening for periodontal disease by the SMT, a combination of two markers would be more accurate in detecting periodontal disease than a single marker. Logistic regression analysis showed that the combination of high occult blood and leukocyte levels showed the highest odds ratio for the presence of both periodontal pockets ≥6 mm and a higher BOP rate. Therefore, the combination of high values of both occult blood and leukocyte levels for the detection of severe periodontitis and gingival inflammation can provide more reliable screening.

There are several limitations in this study. First, this study did not investigate serious dental infections, such as apical periodontitis, which can trigger an inflammatory response in the oral cavity. Since various inflammatory reactions in the oral cavity can affect SMT values, hidden infections could have affected the results. However, these evaluations require a radiographic examination, which is difficult to perform in a community survey with limited facilities. Second, a complete evaluation of all remaining teeth was not conducted, because this study used partial mouth recording of the CPI. The CPI, particularly when used with partial mouth recording, has been reported to underestimate disease prevalence due to site selection bias and limited sensitivity to early lesions [31]. Nonetheless, screening by partial mouth recording of the CPI can identify approximately 85% of those periodontal disease patients detected by full mouth recording [32]; therefore, the use of partial mouth recordings in group examination settings where time is limited may be considered reasonable. Third, the SMT measurement procedure is based on manufacturer-provided information, and there is a lack of independent validation studies or internal assessments of measurement reliability. Further research is warranted to confirm the accuracy and reproducibility of the SMT system in various populations and settings. Fourth, potential confounding variables such as smoking status, oral hygiene behaviors, and systemic health conditions were not assessed or controlled for in the analysis. These factors may influence both salivary biomarkers and periodontal health. In particular, smoking [33] and systemic conditions such as diabetes [34] are known to be associated with both periodontal inflammation and salivary biomarker levels, and their omission may have introduced residual confounding. Future studies should incorporate and adjust for these variables to clarify the independent utility of the SMT. Fifth, the study population consisted exclusively of community-dwelling older adults in Japan. Therefore, the findings may not be generalizable to younger individuals, institutionalized elderly, or populations in other countries with different oral health conditions, health systems, or cultural practices related to dental care.

## 5. Conclusions

The Salivary Multi Test (SMT) may have potential as a supplementary screening tool for periodontal disease in community-dwelling elderly individuals. While individual SMT markers showed modest diagnostic accuracy, their relatively high negative predictive values suggest utility in identifying individuals unlikely to have advanced disease. However, the low specificity and reliance on salivary proxy indicators limit its role as a standalone diagnostic method. Combining multiple markers may improve screening performance, but further validation is needed before broader clinical application.

## Figures and Tables

**Table 1:** Comparisons of values and distribution of SMT items by periodontal status.

SMT Items		Periodontal Pocket		*p*-Value	Gingival Inflammation		*p*-Value
		Without	With		Without	With	
*n*		307	65		279	93	
Occult blood		44.0 (28.0–60.0)	57.0 (43.0–70.0)	<0.001	44.0 (30.0–60.0)	54.0 (35.0–66.0)	0.019
	Normal	170 (89.9)	19 (10.1)	<0.001	155 (82.0)	34 (18.0)	0.002
	Higher	137 (74.9)	46 (25.1)		124 (67.8)	59 (32.2)	
Leukocyte		76.0 (62.0–88.0)	81.0 (68.5–89.0)	0.132	76.0 (62.0–88.0)	80.0 (68.5–87.5)	0.127
	Normal	166 (86.9)	25 (13.1)	0.028	150 (78.5)	41 (21.5)	0.120
	Higher	141 (77.9)	40 (22.1)		129 (71.3)	52 (28.7)	
Protein		73.0 (60.0–85.0)	81.0 (69.5–95.5)	0.002	73.0 (60.0–87.0)	79.0 (64.0–88.5)	0.131
	Normal	169 (88.0)	23 (12.0)	0.004	153 (79.7)	39 (20.3)	0.041
	Higher	138 (76.7)	42 (23.3)		126 (70.0)	54 (30.0)	

Data represents median (first–third quartile) and *n* (%). Periodontal pocket was defined as ≥6 mm. Gingival inflammation was defined as more than 3rd quartile for the BOP rate. Mann–Whitney U test and chi-squared test were performed.

**Table 2:** Sensitivity, specificity, positive predictive value, negative predictive value, and area under the curve for SMT items.

SMT Items	Periodontal Status	Sensitivity	Specificity	PPV	NPV	AUC
Occult blood	Periodontal pocket	71% (60–82)	55% (49–61)	25% (15–36)	90% (87–93)	0.672 (0.605–0.738)
	Gingival inflammation	57% (45–69)	56% (50–61)	32% (21–43)	82% (78–86)	0.588 (0.520–0.656)
Leukocyte	Periodontal pocket	62% (50–74)	54% (48–60)	22% (12–32)	87% (83–91)	0.606 (0.533–0.680)
	Gingival inflammation	56% (44–68)	54% (48–60)	29% (18–40)	79% (74–84)	0.555 (0.488–0.621)
Protein	Periodontal pocket	65% (53–77)	55% (49–61)	23% (13–33)	88% (84–92)	0.638 (0.562–0.714)
	Gingival inflammation	58% (46–70)	55% (49–61)	30% (19–41)	80% (76–84)	0.557 (0.489–0.625)

*n* = 372. PPV, positive predictive value; NPV, negative predictive value; AUC, area under the curve. The cutoff values for SMT items were set to their medians. Values in parentheses represent 95% confidence intervals. Periodontal pocket was defined as ≥6 mm. Gingival inflammation was defined as more than 3rd quartile for the BOP rate.

**Table 3:** Combinations of SMT items and their associations with periodontal status.

Combinations of SMT Items	Periodontal Pocket		*p*-Value	Gingival Inflammation		*p*-Value
	Without	With		Without	With	
*n*	307	65		279	93	
Occult blood and leukocyte						
Occult blood (−) and leukocyte (−)	115 (92.7)	9 (7.3)	0.001	103 (83.1)	21 (16.9)	0.014
Occult blood (−) and leukocyte (+)	55 (84.6)	10 (15.4)		52 (80.0)	13 (20.0)	
Occult blood (+) and leukocyte (−)	51 (76.1)	16 (23.9)		47 (70.1)	20 (29.9)	
Occult blood (+) and leukocyte (+)	86 (74.1)	30 (25.9)		77 (66.4)	39 (33.6)	
Leukocyte and protein						
Leukocyte (−) and protein (−)	115 (89.8)	13 (10.2)	0.015	104 (81.3)	24 (18.8)	0.132
Leukocyte (−) and protein (+)	51 (81.0)	12 (19.0)		46 (73.0)	17 (27.0)	
Leukocyte (+) and protein (−)	54 (84.4)	10 (15.6)		49 (76.6)	15 (23.4)	
Leukocyte (+) and protein (+)	87 (74.4)	30 (25.6)		80 (68.4)	37 (31.6)	
Occult blood and protein						
Occult blood (−) and protein (−)	123 (89.1)	15 (10.9)	<0.001	113 (81.9)	25 (18.1)	0.008
Occult blood (−) and protein (+)	47 (92.2)	4 (7.8)		42 (82.4)	9 (17.6)	
Occult blood (+) and protein (−)	46 (85.2)	8 (14.8)		40 (74.1)	14 (25.9)	
Occult blood (+) and protein (+)	91 (70.5)	38 (29.5)		84 (65.1)	45 (34.9)	

Data represents *n* (%). +: Higher group, −: Normal group. Periodontal pocket was defined as ≥6 mm. Gingival inflammation was defined as more than 3rd quartile for the BOP rate. Chi-squared test was performed.

**Table 4:** Logistic regression analysis for the presence of periodontal pockets ≥6 mm by combinations of SMT items.

Combinations of SMT Items	All, *n*	Cases, *n*	ORs	95% CI	*p*-Value
Occult blood and leukocyte					
Occult blood (−) and leukocyte (−)	124	9	ref		
Occult blood (−) and leukocyte (+)	65	10	2.36	0.90–6.16	0.081
Occult blood (+) and leukocyte (−)	67	16	3.92	1.61–9.54	0.003
Occult blood (+) and leukocyte (+)	116	30	4.55	2.04–10.12	<0.001
Leukocyte and protein					
Leukocyte (−) and protein (−)	128	13	ref		
Leukocyte (−) and protein (+)	63	12	2.16	0.91–5.11	0.081
Leukocyte (+) and protein (−)	64	10	1.85	0.75–4.53	0.181
Leukocyte (+) and protein (+)	117	30	3.04	1.49–6.19	0.002
Occult blood and protein					
Occult blood (−) and Protein (−)	138	15	ref		
Occult blood (−) and Protein (+)	51	4	0.68	0.21–2.17	0.515
Occult blood (+) and Protein (−)	54	8	1.45	0.58–3.67	0.430
Occult blood (+) and Protein (+)	129	38	3.38	1.74–6.55	<0.001

OR: odds ratio, CI: confidence interval. +: Higher group, −: Normal group. Cases were participants with periodontal pockets ≥6 mm. Odds ratios were estimated after adjusting for age and sex.

**Table 5:** Logistic regression analysis for the presence of gingival inflammation by combinations of SMT items.

Combinations of SMT Items	All, *n*	Cases, *n*	ORs	95% CI	*p*-Value
Occult blood and leukocyte					
Occult blood (−) and leukocyte (−)	124	68	ref		
Occult blood (−) and leukocyte (+)	65	30	1.23	0.57–2.65	0.603
Occult blood (+) and leukocyte (−)	67	47	2.13	1.05–4.33	0.036
Occult blood (+) and leukocyte (+)	116	83	2.50	1.36–4.59	0.003
Leukocyte and protein					
Leukocyte (−) and protein (−)	128	70	ref		
Leukocyte (−) and protein (+)	63	37	1.65	0.81–3.37	0.172
Leukocyte (+) and protein (−)	64	45	1.35	0.65–2.81	0.426
Leukocyte (+) and protein (+)	117	76	2.01	1.11–3.63	0.021
Occult blood and protein					
Occult blood (−) and Protein (−)	138	72	ref		
Occult blood (−) and Protein (+)	51	26	0.96	0.42–2.23	0.929
Occult blood (+) and Protein (−)	54	35	1.59	0.75–3.35	0.228
Occult blood (+) and Protein (+)	129	95	2.47	1.40–4.35	0.002

OR: odds ratio, CI: confidence interval. +: Higher group, −: Normal group. Cases were participants with gingival inflammation. Odds ratios were estimated after adjusting for age and sex.

## Data Availability

A request for data disclosure will be granted at the discretion of the Facility Ethics Committee.

## Abbreviations

SMT	Salivary Multi Test
BOP	Bleeding on probing
CPI	Community Periodontal Index
PPV	Positive predictive value
NPV	Negative predictive value
ROC	Receiver operating characteristic
AUC	Area under the curve
